# Numerical Investigation of Photo-Generated Carrier Recombination Dynamics on the Device Characteristics for the Perovskite/Carbon Nitride Absorber-Layer Solar Cell

**DOI:** 10.3390/nano12224012

**Published:** 2022-11-15

**Authors:** Faisal Saeed, Muhammad Haseeb Khan, Haider Ali Tauqeer, Asfand Haroon, Asad Idrees, Syed Mzhar Shehrazi, Lukas Prokop, Vojtech Blazek, Stanislav Misak, Nasim Ullah

**Affiliations:** 1 Functional Materials and Optoelectronic Devices (FMOD) Lab, Department of Physics, Lahore University of Management Sciences (LUMS), Lahore 54792, Pakistan; 2 Department of Electrical Engineering, Lahore University of Management Sciences (LUMS), Lahore 54792, Pakistan; 3 Department of Electrical Engineering, University of Engineering and Technology Lahore, Lahore 39161, Pakistan; 4 Department of Electrical Engineering, University of Lahore, Lahore 54590, Pakistan; 5 ENET Centre, VSB—Technical University of Ostrava, 708 00 Ostrava, Czech Republic; 6 Department of Electrical Engineering, College of Engineering, Taif University, P.O. Box 11099, Taif 21944, Saudi Arabia

**Keywords:** double absorber layer solar cell, recombination, numerical investigation

## Abstract

The nitrogenated holey two-dimensional carbon nitride ($C_{2}N$) has been efficaciously utilized in the fabrication of transistors, sensors, and batteries in recent years, but lacks application in the photovoltaic industry. The $C_{2}N$ possesses favorable optoelectronic properties. To investigate its potential feasibility for solar cells (as either an absorber layer/interface layer), we foremost detailed the numerical modeling of the double-absorber-layer–methyl ammonium lead iodide (${\mathrm{CH}}_{3}{\mathrm{NH}}_{3}{\mathrm{PbI}}_{3}$) –carbon nitride ($C_{2}N$) layer solar cell and subsequently provided in-depth insight into the active-layer-associated recombination losses limiting the efficiency (η) of the solar cell. Under the recombination kinetics phenomena, we explored the influence of radiative recombination, Auger recombination, Shockley Read Hall recombination, the energy distribution of defects, Band Tail recombination (Hoping Model), Gaussian distribution, and metastable defect states, including single-donor (0/+), single-acceptor (−/0), double-donor (0/+/2+), double-acceptor (2/−/0−), and the interface-layer defects on the output characteristics of the solar cell. Setting the defect (or trap) density to $10^{15}{\mathrm{cm}}^{-3}$ with a uniform energy distribution of defects for all layers, we achieved an η of 24.16%. A considerable enhancement in power-conversion efficiency ( η~27%) was perceived as we reduced the trap density to $10^{14}{\mathrm{cm}}^{-3}$ for the absorber layers. Furthermore, it was observed that, for the absorber layer with double-donor defect states, the active layer should be carefully synthesized to reduce crystal-order defects to keep the total defect density as low as $10^{17}{\mathrm{cm}}^{-3}$ to achieve efficient device characteristics.

## 1. Introduction

At present, photovoltaic modules based on wafer-based crystalline silicon solar cells account for >90% of the global photovoltaic market [1,2,3,4,5]. Laudable enhancements in power conversion efficiency (η) have been experienced for this technology over the last few years, leading to thin film, tandem, and various lab-based architectures [6,7,8,9,10,11,12,13]. Additionally, the manufacturing process of crystalline-based solar modules requires expensive materials and high production costs. Given that, double-absorber-layer (DAL) solar cells, rivaling the tandem solar cells, can be considered a promising candidate, among emerging photovoltaic technology, achieving a high device performance while cutting costs [14].

Despite perceptible advancements in tandem solar cells (perovskite/silicon tandem solar cells that have been reported recently, with an impressive η~29.15% [15]), the ease of fabricating DAL solar cells is one of the major advantages reducing complexity in tuning two-terminal tandem solar cells or managing the four-terminal tandem device circuitry. Besides this, DAL solar cells can be characterized by low production costs, compared to tandem solar cells, making them a potential candidate for the future of the photovoltaic industry [16].

The composition of DAL solar cells requires the absorber layers (or active layers) to have an almost similar lattice structure [16]. The active layers can be chalcogenides, perovskites, polymers, or other organic/inorganic layers. In such an architecture, the absorber layer with varied energy band gaps, which form the junctions sequentially, harness photo-generated power from their respective portions of the solar spectrum. This makes the multi-junction devices efficient and comparable to single-junction ones. However, the success of such DAL devices lies in the current matching of the active-layer junctions and efficient recombination of photo-generated carriers from the adjacent junctions.

To date, limited research has reported on the theoretical modeling/fabrication of DAL solar cells. Ho Yeon, Deuk, et al. [17] reported a 4% efficient PbS/Cds solar cell fabricated by chemical bath deposition. Ahmad, Faiz et al. [14] theoretically proposed an optical modeling of a ${\mathrm{CuIn}}_{1-\xi_{1}}{\mathrm{Ga}}_{\xi_{1}}{\mathrm{Se}}_{2}/{\mathrm{Cu}}_{2}\mathrm{ZnSn}\left(S_{\xi_{2}}{\mathrm{Se}}_{1-\xi_{2}}\right)$ absorber layer solar cell with an impressive η~34.45%. AlZoubi, Tariq, et al. [18] detailed a numerical modeling of a CZTS/Si-based active layer solar cell with η ~29.15%. Maurya, K et al. [19] computationally detailed a >35% efficient thin-film device based on an ${\mathrm{Sb}}_{2}{\mathrm{Se}}_{3}$/CZTS absorber layer. S Yasin et al. [16] recently detailed a $C_{2}N/{\mathrm{FASnI}}_{3}$ absorber layer solar cell with η ~25.15%, keeping the trap density at $10^{14}{\mathrm{cm}}^{-3}$.

Here, we proposed a novel structured DAL, employing metal halide perovskite and Carbon Nitride ($C_{2}N$) as the absorber layers. Carbon Nitride ($C_{2}N$) is a 2D material with a structural composition similar to graphene, with a wider energy band gap ~ 1.8 eV, and a higher optical absorption in the visible spectrum. $C_{2}N$ has been used for photocatalysis, and in the fabrication of field effect transistors FETs, biosensors, batteries, and hydrogen storing [20,21,22,23]. $C_{2}N$ material has favorable properties for use as a primary absorber for photovoltaic applications. It has been numerically explored for photovoltaic cell modeling but has not yet been reported in the literature. We, therefore, focused on a defect-based study for the absorber layers, to comprehensively investigate the proposed active layered structure solar cell favorability for future thin-film photovoltaic applications.

## 2. Numerical Modeling and Material Parameters

**Figure 1:**
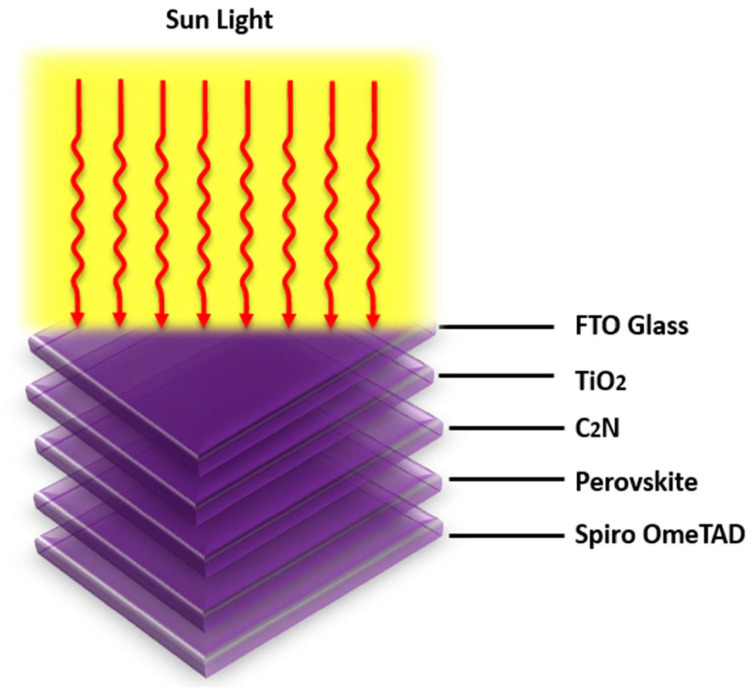
Proposed double-absorber-layer solar cell-layer structure.

The proposed solar cell is composed of FTO/${\mathrm{TiO}}_{2}$/$C_{2}N/{\mathrm{CH}}_{3}{\mathrm{NH}}_{3}{\mathrm{PbI}}_{3}$/SpiroOmeTAD/Au-back metal contact (see Figure 1) and was numerically modeled and investigated in SCAPS-1D, which is based on three coupled semiconductor differential equations: Poisson’s equation, and the continuity equations for electrons and holes (1) [7]. The material parameters for the simulation are enlisted in Appendix A Table A1. The SCAPS numerically evaluates the steady-state solution of these equations with appropriate boundary conditions [24,25,26,27].

(1) $$\{\begin{array}{l} \;\nabla^{2}V\;\left(x\right)=\frac{q}{\varepsilon }\left[p\left(x\right)-n\left(x\right)+N_{D}{}^{+}\left(x\right)-N_{A}{}^{-}\left(x\right)+N_{tr}{}^{\pm }\right]\\ \frac{\partial p\left(n\right)}{\partial t}=G_{p}\left(x\right)-\frac{p_{n}-p_{no}}{\tau_{p}}-p_{n}\mu_{p}\frac{d\xi }{dx}+\mu_{p}\xi \frac{dp_{n}}{dx}+D_{p}\frac{d^{2}p_{n}}{dx^{2}}\\ \frac{dn_{p}}{dt}=G_{n}\left(x\right)-\frac{n_{p}-n_{po}}{\tau_{n}}-n_{p}\mu_{n}\frac{d\xi }{dx}+\mu_{n}\xi \frac{dn_{p}}{dx}+D_{n}\frac{d^{2}n_{p}}{dx^{2}} \end{array}\;$$

where $\nabla^{2}V$ is the electrostatic potential, *q* is the electronic charge, p(x) and n(x) are the position-dependent hole and electron concentration, $N_{D}{}^{+}\left(x\right)$ and $N_{A}{}^{-}\left(x\right)$ is the position-dependent ionized dopant and acceptor concentration, $N_{tr}{}^{\pm }$ is the shallow/bulk trap (or defect) carrier concentration. The electron–hole pair generation, G(x) in the absorber layer is a result of incident photon flux ($N_{phot}$) of wavelength (λ), at each position (x) within the layer, and follows the mathematical relation (2) [28]. $\lambda_{min}$,$\;\lambda_{max}$ are the minimum and maximum wavelengths of the incoming solar spectrum.

(2) $$G\left(\lambda ,x\right)=\alpha \left(\left(\lambda ,x\right)\right).N_{phot}\left(\lambda ,x\right)=\int_{\lambda_{min}\;}^{\lambda_{max}}G\left(\lambda ,x\right)\;d\lambda =\int_{\lambda_{min}\;}^{\lambda_{max}}\alpha \left(\left(\lambda ,x\right)\right).N_{phot}\left(\lambda ,x\right)d\lambda$$

where, in

(3) $$N_{phot}\left(\lambda ,x\right)=N_{phot0}\left(\lambda \right).T_{front}\left(\lambda \right).\exp\left(-x\alpha \left(\lambda \right)\right).\frac{1+R_{back}\left(\lambda \right)\exp\left(-2\left(d-x\right)\alpha \left(\lambda \right)\right)}{1-R_{back}\left(\lambda \right)R_{int}\exp\left(-2d\alpha \left(\lambda \right)\right)}$$

In the above equation, $T_{front}\left(\lambda \right)$ is the transmission at the front contact (wavelength-dependent), $R_{back}\left(\lambda \right)$ is the reflection at the back contact (wavelength-dependent), $R_{int}$ is the internal reflection at the front contact, and *d* is the layer thickness.

Further, we employed one of the four SCAPS inbuilt optical absorption (α) models following the expression (4) [28]. In this model energy band gap, $\left(E_{g}\right)$ follows the square root law and α = 0 if the incident photon energy is $<E_{g}$.

(4) $$\alpha \left(hv\right)=\left(\alpha_{o}+\frac{\beta_{o}E_{g}}{hv}\right)\sqrt{\frac{hv}{E_{g}}-1}\;$$

**Figure 2:**
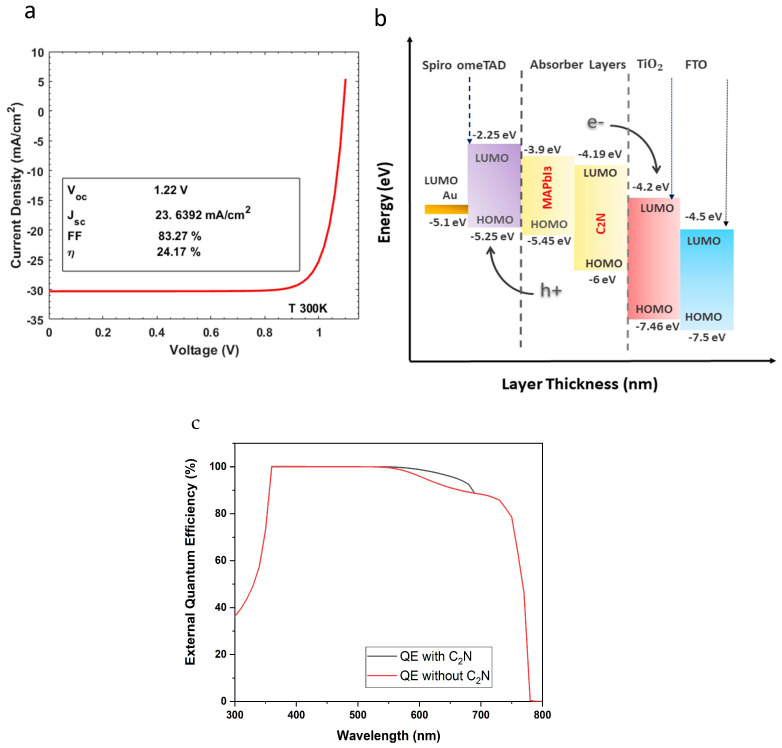
The (**a**) current density–voltage characteristics of the solar, (**b**) energy level diagram of the double-absorber-layer solar cell, (**c**) external quantum efficiency of the solar cell with and without out carbon nitride.

The device with only the perovskite absorber layer demonstrated an η of 23.83%, an open-circuit voltage ($V_{oc}$) of 1.22 V, a short circuit current density ($J_{sc}$) of 23.3418 $\mathrm{mA}/{\mathrm{cm}}^{2},\;\mathrm{and}$ a fill factor (FF) of 83.18%. The devices with optimized thicknesses with perovskite/$C_{2}N$ demonstrated an increased η of 24.17%, an open-circuit voltage ($V_{oc}$) of 1.22 V, a short circuit current density ($J_{sc}$) of 23.6392 $\mathrm{mA}/{\mathrm{cm}}^{2},$ and a fill factor (FF) of 83.27%. The current density–voltage curve of the DAL solar, under standard illumination conditions, is depicted in Figure 2a. Figure 2b illustrates the energy level diagram of the solar cell. Furthermore, the external quantum efficiency of the solar cell with $C_{2}N$ (see Figure 2c) remained at > 90% for the near-ultraviolet region, (360 nm ≤ incident light ≥ 360 nm or photon energy, $E_{p}$~3.44 eV) to the major part of the visible-light spectrum (incident light wavelengths ≤360 nm or $E_{p}$~3.44 eV), clearly showing a better quantum efficiency response than a single-absorber layer. It should be noted that, in further sections, defects are simultaneously introduced/changed in perovskite/carbon nitride absorber layers for investigation into the impact of the recombination phenomenon on device performance.

## 3. Results and Discussion

### 3.1. Influence of Recombination on Device Performance

**Figure 3:**
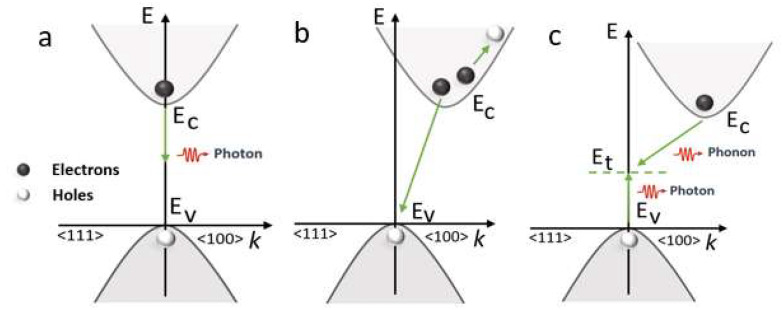
Photogenerated carrier recombination mechanism. (**a**) Radiative recombination, (**b**) Auger recombination and (**c**) SRH recombination using energy (E) momentum (K) diagram.

**Figure 4:**
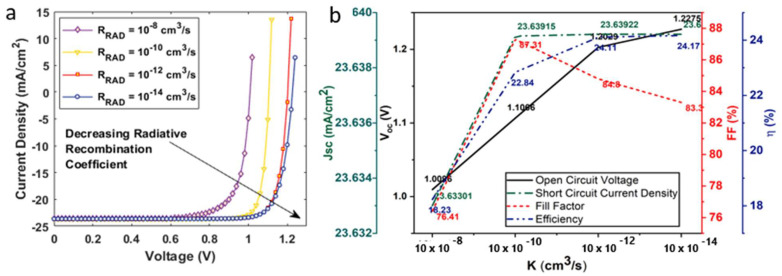
(**a**) Current density–voltage characteristics of a solar cell under radiative recombination; (**b**) influence of radiative recombination on the open circuit voltage, short circuit current density, fill factor, and efficiency of the solar cell.

**Figure 5:**
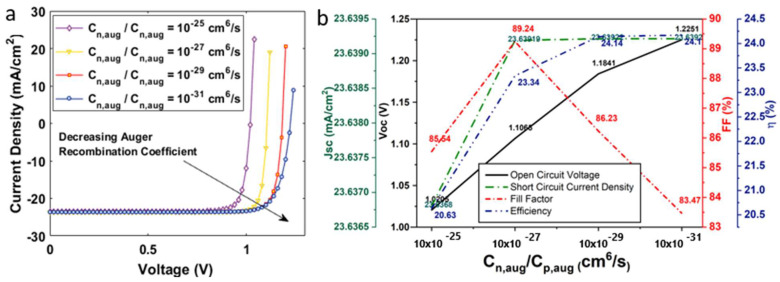
(**a**) Current density–voltage characteristics of a solar cell under auger recombination; (**b**) influence of Auger recombination coefficient on the open circuit voltage, short circuit current density, fill factor, and efficiency of the solar cell.

**Figure 6:**
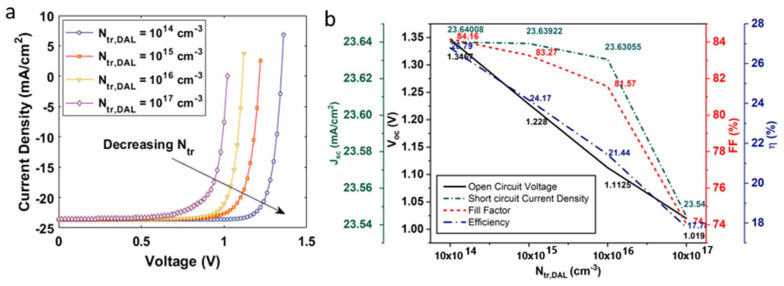
(**a**) Current density–voltage characteristics of a solar cell under auger recombination; (**b**) influence of defect density-assisted SRH recombination on the open circuit voltage, short circuit current density, fill factor, and efficiency of the solar cell.

Ideally, photovoltaic material has a higher absorption coefficient to effectively harvest incident solar energy photons and convert them into free charge carriers. However, recombination losses in solar cells are inevitable due to material defects [29]. Recombination losses affect the collection current, as well as the forward-bias injection current. This directly influences the short-circuit current density and open-circuit voltage of the solar cell, thereby limiting the fill factor and efficiency of the solar cell [30]. Recombination mechanisms considered in this investigation for the ${\mathrm{CH}}_{3}{\mathrm{NH}}_{3}{\mathrm{PbI}}_{3}$/$C_{2}N$ absorber layer include radiative recombination ($R_{Rad}$), Auger ($R_{Aug}$) and Shockley Read Hall recombination ($R_{SRH}$), following expression (5). More insight into the recombination phenomenon is provided in Figure 3.

(5) $$\{\begin{array}{c} R=R_{Rad}+R_{Aug}+R_{SRH\;}\;\\ R_{Rad}=K\;\left(np-n_{i}^{2}\right)\;\\ \;R_{Aug}=\left(C_{n,aug}n+C_{n,aug}p\right)\left(np-n_{i}^{2}\right)\;\\ R_{SRH}=\frac{\left(np-n_{i}^{2}\right)}{\tau_{p}\;\left(n+ni\exp\left(\frac{E_{t}-E_{i}}{kT}\right)\right)+\tau_{n}\;\left(p+ni\exp\left(\frac{E_{i}-E_{t}}{kT}\right)\right)}\; \end{array}$$

where K is the radiative recombination coefficient, $C_{n}^{A}\;\left(C_{p}^{A}\right)$ is the Auger electron (hole) recombination coefficient, n (p) is the electron (hole) carrier concentration, $\tau_{n}$ ($\tau_{p}$) is the electron (hole) carrier lifetime, $E_{i}$ is the intrinsic energy level, $E_{t}$ is the trap energy level, and T is the temperature at room temperature. The K factor for ${\mathrm{CH}}_{3}{\mathrm{NH}}_{3}{\mathrm{PbI}}_{3},$ as calculated by the first principles, is reported in the range of (0.5–1.5)$\;\times \;10^{-9}\;\left({\mathrm{cm}}^{3}/s\right)$. The range of $C_{n}^{A}\;\left(C_{p}^{A}The\right)$ factor for ${\mathrm{CH}}_{3}{\mathrm{NH}}_{3}{\mathrm{PbI}}_{3}$ perovskite material, as evaluated from time- and excitation-energy-dependent photoluminescence spectroscopy, has been reported to lie between 1.8 and 3.7. The point defect study on $C_{2}N$ confirmed that such materials exhibit both a direct and indirect energy bandgap nature. We therefore set similar K and $C_{n}^{A}\;\left(C_{p}^{A}The\right)$ factors for both the absorber layers. The device’s current density voltage characteristics under radiative, Auger, and SRH recombination are shown in Figure 4a, Figure 5a and Figure 6a, respectively.

To investigate the impact of radiative recombination, we varied the K factor in the range of $10^{-8}-10^{-14}\;\left({\mathrm{cm}}^{3}/s\right)$ (see Figure 4b). An increased value of K has an adverse effect on the output characteristics of the solar cell. It was observed that the device demonstrated a maximum η~24.17%, $V_{oc}\;\sim \;1.22\;V$, and $J_{sc}\;\sim 23.64\;\mathrm{mA}/{\mathrm{cm}}^{2}$ at K = $10^{-14}{\;\mathrm{cm}}^{3}/s,$ as illustrated in Figure 4. The highest FF~87.31% was obtained at K = $10^{-10}$ ${\mathrm{cm}}^{3}/s$. The device’s η fell to ~16% as we increased the K to $10^{-8}\;cm^{3}/s$. To investigate the influence of $R_{Aug}$ on device performance, we varied the $C_{n,aug}\left(C_{p,aug}\right)$ at $10^{-25}-10^{-31}\;\left({\mathrm{cm}}^{6}/s\right)$ (see Figure 5). The device demonstrated a maximum η at $C_{n,aug}$ = $10^{-31}{\;\mathrm{cm}}^{6}/s$ and a minimum $C_{n,aug}$ = $10^{-25}{\;\mathrm{cm}}^{6}/s$. To analyze $R_{SRH}$, we employed the trap density model as it has been elaborated in previous studies. The trap density for the double-absorber layer, $N_{tr,DAL}$ was within the range $10^{14}-10^{17}{\;\mathrm{cm}}^{-3}$ (see Figure 6). As discussed earlier, the device was simulated with a defect density of $10^{15}{\;\mathrm{cm}}^{-3}$. On decreasing the defect density to $10^{14}{\;\mathrm{cm}}^{-3}$, the device demonstrated a maximum η~26.18%, $V_{oc}\;\sim \;1.34\;V$, and $J_{sc}\;\sim 26.79\;\mathrm{mA}/{\mathrm{cm}}^{2}$, and the device η reduced to ~17%, including other device parameters, as we increased the defect density to $10^{17}{\;\mathrm{cm}}^{-3}$.

### 3.2. Influence of Energy Distribution of Defects on the Device Performance

**Figure 7:**
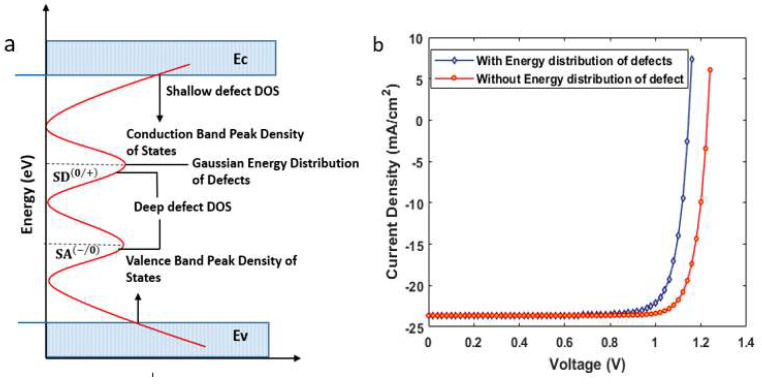
(**a**) Density of state (DOS) of the semiconductor material; (**b**) current density–voltage characteristics for the solar cell with and without adoption of energy distribution of defects.

In organic–inorganic absorber layers, the energy distribution of defect modeling is imperative to accurately model the device. The total defect density of state (DOS) in the absorber layer is assumed to comprise shallow level defects, modeled by exponentially decaying conduction or valence band tail states, and deep-level defects modeled by Gaussian distribution in the mid-gaps (see Figure 7a) [31,32]. The Gaussian conduction/valence band tail state, and energy distribution in the SCAPS environment follow the mathematical relation (6–8) [28] where $E_{t}$ is the tap energy level, $E_{c}$ is the characteristic energy, $w_{G}$ is the width of Gaussian energy distribution, $w_{t}$ is the width of tail-like distribution, $N_{t}\left(E\right)$ is the defect density in ${\mathrm{cm}}^{-3}/\mathrm{eV}$, and $N_{peak}$ is the peak density of the energy distribution. Band tailing hampers the mobility of photo-generated carriers to a great extent by trapping and de-trapping. The $w_{G}$ ($or\;w_{t}$) is related to the degree of disorder in crystals [33]. Experimentally reported values for perovskite material are in the range of 15−63 (meV) [34].

However, we kept the $w_{G}$ as 0.564 eV, while $w_{t}$ = 0.1 eV for the absorber layers. The $N_{peak}$ in all the above-mentioned energy distributions is $10^{15}\;\left(1/\mathrm{eV}/{\mathrm{cm}}^{3}\right)$, with total defect density states of $10^{15}{\;\mathrm{cm}}^{-3}.$

(6) $$\overset{Gaussian\;Energy\;Distribution}{\overbrace{\begin{array}{c} Range=\left[E_{t}-w_{G}\frac{E_{c}}{2};E_{t}+w_{G}\frac{E_{c}}{2}\right],\;N_{t}\left(E\right)=N_{peak}\times \exp\left[-{\left(\frac{E-E_{t}\;}{E_{c}}\right)}^{2}\right] \end{array}}}$$

(7) $$\overset{Conduction\;Band\;Energy\;Distribution\;}{\overbrace{\begin{array}{c} Range=\left[E_{t}-w_{t}E_{c};E_{t}\right],\;N_{t}\left(E\right)=N_{peak}\times \exp\left[\frac{E-E_{t}}{E_{c}}\right] \end{array}}}$$

(8) $$\overset{Valence\;Band\;Energy\;Distribution}{\overbrace{\begin{array}{c} Range=\left[E_{t};E_{t}+w_{t}E_{c}\right],\;N_{t}\left(E\right)=N_{peak}\times \exp-\left[\frac{E-E_{t}}{E_{c}}\right] \end{array}}}$$

The device was simulated with the above modeling, and current density vs. voltage characteristics are shown in Figure 7b. the device η was decreased to 22.12% from 24.17%, $V_{oc}$ to ~1.14 V, $J_{sc}$ to ~23.634 $\mathrm{mA}/{\mathrm{cm}}^{2}$, and FF~81 as can be observed in the figure. The current density curve under both conditions is summarized in Table 1.

### 3.3. Influence of Metastable Defects on the Device Performance

**Figure 8:**
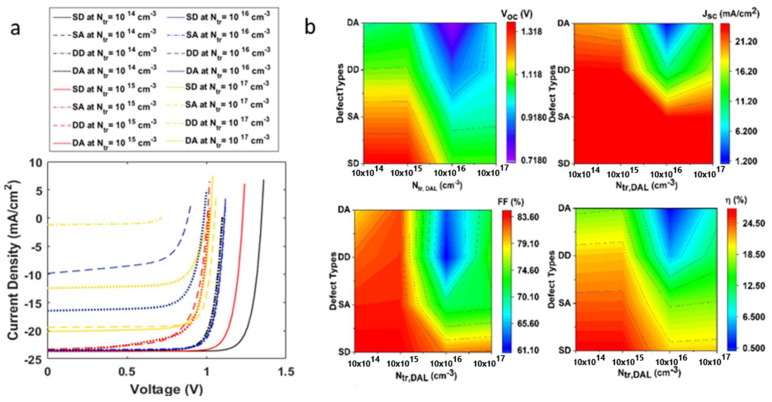
(**a**) Current–voltage characteristics of a solar cell on inclusion of metastable defect states with varying trap densities (**b**) Influence of metastable defect states, single donor (SD), single acceptor (SA), double donor (DD), and double acceptor (DA), at varying defect densities, on open-circuit voltage, short-circuit current density, fill factor and efficiency of the solar cell.

In this section, we investigated the impact of metastable defect transition on the absorber layers. In ${\mathrm{CH}}_{3}{\mathrm{NH}}_{3}{\mathrm{PbI}}_{3}$, halide ion segregation requires the migration of halide ions, which is a defect-driven process resulting in halide vacancy defects [35]. For the absorber layer, we induced double-vacancy defects, including a single donor ($SD^{\left(0/+\right)}$), double acceptor ($SD^{\left(-/0\right)}$), double donor ($DD^{\left(0/+/2/+\right)}$), and double acceptor ($DA^{\left(2/-/0/-\right)}$), at varying total trap densities. The single (double)-donor defect states per unit of volume are concentrated closer to the conduction band edge, while single (double) acceptors are concentrated closer to the valence band [36,37]. The impact of metastable defects on device output characteristics at varied trap densities ($10^{14}-10^{17}{\;\mathrm{cm}}^{-3})$ is summarized in Table 2, Table 3, Table 4 and Table 5. It was observed that single-donor defects affected the device output characteristics the least. However, the $DD^{\left(0/+/2/+\right)}$ defect considerably affected solar cell performance in all conditions. The current density voltage characteristics for metastable state defects are depicted in Figure 8a, while Figure 8b provides more insight into the results.

### 3.4. Influence of Interface Defects on the Device Performance

**Figure 9:**
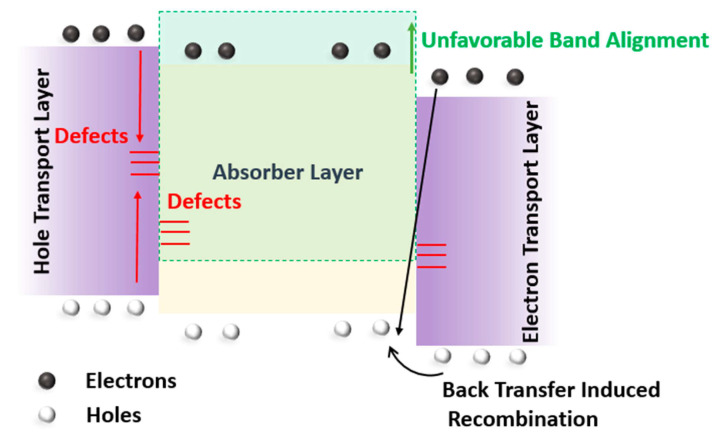
Illustration of interface defects mechanism in a solar cell.

Interfacial recombination plays a significant role in determining the performance of the solar cell. Interface defects emerge due to recombination centers at the interface of the absorber material/hole-transport layer (or electron-transport layer) [38]. These recombination centers can be present inside the absorber layer or hole-transport layer (or electron-transport layer), at the interface. Other reasons for interface defects can be an unfavorable HTL/absorber layer (or absorber layer/ETL), band alignment, and back-transfer-induced recombination [39], as illustrated in Figure 9.

**Figure 10:**
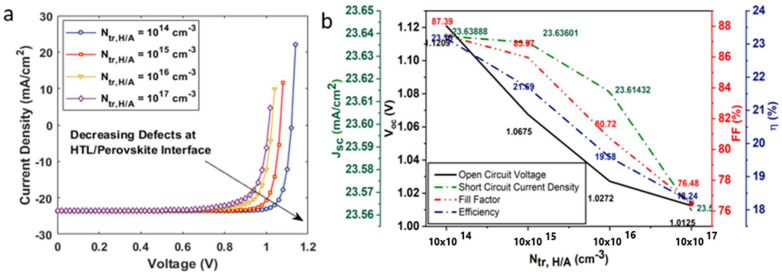
(**a**) Current voltage characteristics of solar on inclusion of interface defects at hole transport layer/absorber layer; (**b**) influence of interface defects on open-circuit voltage, short-circuit current density, fill factor and efficiency of the solar cell.

**Figure 11:**
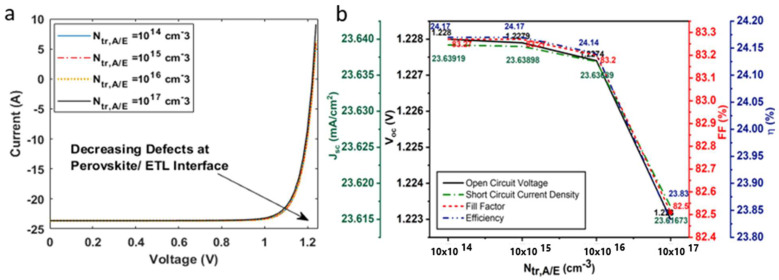
(**a**) Current voltage characteristics of solar on inclusion of interface defects at hole transport layer/absorber layer; (**b**) influence of interface defects on open-circuit voltage, short-circuit current density, fill factor, and efficiency of the solar cell.

The current density–voltage curve shown in Figure 10a illustrates the effect of varying interface defect densities at the hole transport layer/absorber layer interface, $N_{tr,H/A}$. The $N_{tr,H/A}$ was varied in the range of $10^{14}-10^{17}\;({\mathrm{cm}}^{-3}$). Interface defects considerably affected the device power conversion efficiency of the solar cell, in comparison to $J_{sc},V_{oc},\;FF$, as can be observed from Figure 10b. At $N_{tr,H/A}$ = $10^{14}{\;\mathrm{cm}}^{-3}$, the device η was 23.19% and was decreased to 18.24% at $N_{tr,H/A}$ = $10^{14}{\;\mathrm{cm}}^{-3}$, indicating high recombination at the interface (Figure 11). Similarly, interface defect density at the absorber layer/electron transport layer interface, $N_{tr,A/E}$. was also varied in the range of $10^{14}-10^{17}\;({\mathrm{cm}}^{-3}$). Unlike $N_{tr,H/A}$, $N_{tr,A/E}$ did not significantly affect the device performance. The device η, $V_{oc}$, FF, and $J_{sc}$ retained their initial optimized values at $N_{tr,A/E}$ of $10^{14}-10^{16}{\;\mathrm{cm}}^{-3}$. However, η fell slightly to 23.83%, from 24.17%, on a further increase in interface defects.

## 4. Conclusions

The combination of two absorber layers, carbon nitride and a perovskite absorber layer, aided in the utilization of a broader range of solar spectrum for solar energy conversion. The device demonstrated high efficiency (24.17%), open-circuit voltage (1.2 V), and fill factor (83.2%), with a uniform DOS energy bandgap. However, the focus was on the computational investigation of dominant recombination mechanisms associated with the absorber layer, to accurately investigate the device performance. The device η remained > 16% under higher radiative, auger coefficient, and trap-assisted recombination. Thereafter, we modeled the Gaussian distribution energy profile for shallow-level defects and Urbach tail states for shallow-level defects. This resulted in device efficiency falling to 22.14%. Further, various double-vacancy-based metastable defect states were induced in the absorber layer. It was observed that double-donor metastable defects highly affected the performance of the solar cell. Finally, we also investigated the influence of interface defects. It was revealed that. for the proposed device architecture, increased defects in the HTL/absorber layer dominantly affected the device performance, instead of absorber-layer/ETL interface defects.

## Figures and Tables

**Table 1:** Device output characteristics with adoption of energy distribution of defects.

	*V_oc_* (V)	*J_sc_* (mA/cm^2^)	FF (%)	*η* (%)
Without Energy Distribution of Defects	1.22	23.692	83.92	24.17
With Energy Distribution of Defects	1.14	23.634	81.01	22.12

**Table 2:** Defect state effect on output characteristics of solar cell at trap density of $10^{17}$ ${\mathrm{cm}}^{-3}$.

	*V_oc_* (V)	*J_sc_* (mA/cm^2^)	FF (%)	*η* (%)
Single Donor	1.3467	23.64038	84.16	26.79
Single Acceptor	1.3467	23.64012	84.16	26.79
Double Donor	1.0996	23.62985	81.09	21.07
Double Acceptor	1.0997	23.62824	81.71	21.23

**Table 3:** Defect state effect on output characteristics of solar cell at trap density $10^{15}$ ${\mathrm{cm}}^{-3}$.

	*V_oc_* (V)	*J_sc_* (mA/cm^2^)	FF (%)	*η* (%)
Single Donor	1.228	23.63991	83.23	24.16
Single Acceptor	1.228	23.63899	83.29	24.18
Double Donor	1.005	23.29016	70.79	16.58
Double Acceptor	1.0168	23.42961	71.89	17.12

**Table 4:** Defect state effect on output characteristics of solar cell at trap density $10^{16}$ ${\mathrm{cm}}^{-3}$.

	*V_oc_* (V)	*J_sc_* (mA/cm^2^)	FF (%)	*η* (%)
Single Donor	1.1131	23.51052	81.76	21.4
Single Acceptor	1.115	23.45095	82.84	21.66
Double Donor	0.8839	9.829793	61.15	5.31
Double Acceptor	0.9845	16.46489	74.39	12.06

**Table 5:** Double donor defect state effect on output characteristics of solar cell.

	*V_oc_* (V)	*J_sc_* (mA/cm^2^)	FF (%)	*η* (%)
Single Donor	1.0251	20.1563	78.86	16.29
Single Acceptor	1.0525	19.40046	82.35	16.82
Double Donor	0.7186	1.200655	63.14	0.54
Double Acceptor	1.0141	12.45877	72.17	9.12

## Data Availability

Not applicable.

## Nomenclature

η	Efficiency
$V_{oc}$	Open Circuit Voltage
$J_{sc}$	Short-Circuit Current Density
FF	Fill Factor
HTL	Hole transport Layer
ETL	Electron Transport Layer
DAL	Double Absorber Layer
λ	Wavelength
p(x)	Position-dependent hole concentration
n(x)	Position-dependent electron concentration
$N_{tr}{}^{\pm }$	Shallow/bulk carrier concentration
G	Electron–hole pair generation
$\lambda_{min}$	Minimum wavelength
$\lambda_{max}$	Maximum wavelength
$T_{front}\left(\lambda \right)$	Transmission at front contact dependent on wavelength
$R_{back}\left(\lambda \right)$	Reflection at back contact dependent on wavelength
$R_{int}\left(\lambda \right)$	Internal reflection at front contact
D	Layer thickness
$E_{g}$	Energy bandgap
α	Optical absorption
$E_{p}$	Photon energy
$R_{RAD}$	Radiative recombination
$R_{AUG}$	Auger recombination
$R_{SRH}$	Schokley Read hall recombination
K	Radiative recombination coefficient
$C_{n,aug}$	Auger–electron recombination coefficient
$C_{p,aug}$	Auger–hole recombination coefficient
n	Electron concentration
p	Hole concentration
$n_{i}$	Intrinsic-carrier concentration
$\tau_{n}$	Electron-carrier lifetime
$\tau_{p}$	Hole-carrier lifetime
$N_{tr,DAL}$	Trap density of double absorber layer
$E_{c}$	Characteristic energy
$w_{g}$	Width of Gaussian energy distribution
$w_{t}$	Width of tail-like distribution
$N_{peak}$	Peak density of the distribution
$E_{t}$	Energy trap level
$E_{i}$	Intrinsic energy level
$SD^{\left(0/+\right)}$	Single-donor defect states
$DD^{\left(0/+/2/+\right)}$	Double-donor defect states
$SA^{\left(-/0\right)}$	Single-acceptor defect states
$DA^{\left(2/-/0/-\right)}$	Double-acceptor defect states
$N_{tr,H/A}$	Interface defect density at hole transport layer/absorber layer interface
$N_{tr,A/E}$	Interface defect density at absorber layer/electron transport layer interface

## Appendix A. Layer Parameters for Numerical Modeling of Double Absorber Layer Solar Cell

**Table A1:** Input layer parameters for numerical modeling of double-absorber-layer solar cell. The material parameters were adopted from the literature [16,40], as well as being self-ascribed.

Parameters	HTL	Perovskite	Carbon Nitride	ETL	FTO
Thickness (nm)	150	600	320	30	600
Energy bandgap, $E_{g}$ (eV)	3.04	1.55	1.8	3.2	3.5
Electron affinity, χ (eV)	2.2	3.9	4.2	4.1	4.0
Relative -ermittivity, $\varepsilon_{r}$	3.0	6.5	4.5	9	9
Density of states at conduction band, $N_{c}$ (cm^−3^)	$2.5\times 10^{19}$	$2.5\times 10^{19}$	$2.5\times 10^{19}$	$2.5\times 10^{19}$	$2.5\times 10^{19}$
Density of states valance band, $N_{v}$ (cm^−3^)	$2.5\times 10^{19}$	$2.5\times 10^{19}$	$2.5\times 10^{19}$	$2.5\times 10^{19}$	$2.5\times 10^{19}$
Electron mobility, $\mu_{e}$ (cm^2^/Vs)	1.0 × 10^−4^	2	12	$5.0\times 10^{-2}$	330
Hole mobility, $\mu_{h}$ (cm^2^/Vs)	$1.0\times 10^{-4}$	2	20	$5.0\times 10^{-2}$	50
Acceptor concentration, $N_{a}$ (cm^−3^)	$1.0\times 10^{18}$	$2.0\times 10^{13}$	0	0	0
Donor concentration, $N_{d}$ (cm^−3^)	0	$3.0\times 10^{13}$	$1.0\times 10^{13}$	$1.0\times 10^{18}$	$2.0\times 10^{19}$
Defect density, $N_{t}$ (cm^−3^)	$1.0\times 10^{15}$	$1.0\times 10^{15}$	$1.0\times 10^{15}$	$1.0\times 10^{15}$	$1.0\times 10^{15}$
